# Remodeling of gut bacteriome and virome in acute retinal necrosis: expansion of *Enterobacteriaceae*-related taxa

**DOI:** 10.3389/fmicb.2026.1848524

**Published:** 2026-06-29

**Authors:** Mingzhu Liu, Tao Liu, Siyan Jin, Pei Liu, Xia Wang, Xiaoli Liu

**Affiliations:** Ophthalmologic Center of the Second Hospital, Jilin University, Changchun, China

**Keywords:** acute retinal necrosis, cross-kingdom associations, *Enterobacteriaceae*, gut bacteriome, gut phages

## Abstract

**Background:**

This study was designed to examine the alterations in the gut bacteriome and virome of patients with acute retinal necrosis (ARN), and to explore potential cross-kingdom microbial associations.

**Methods:**

The gut virome and bacteriome of 10 patients with new-onset ARN and 10 age- and sex-matched healthy individuals (N) were profiled using viral metagenomics and 16S rRNA sequencing, respectively.

**Results:**

The gut bacteriome in ARN patients was significantly altered, with *Enterobacteriaceae_A* increased at the family level. Genus-level analysis further described higher relative abundances of opportunistic pathogens, such as *Escherichia* and *Klebsiella*, alongside lower relative abundances of commensal anaerobes, including *Fusicatenibacter* and *Anaerobutyricum*. Exploratory clinical association analysis suggested a positive association between *Klebsiella* and intraocular pressure, while *Fusicatenibacter* tended to be further reduced in patients with vasculitis involving the major retinal arteries. Predicted bacterial functional profiling indicated an enrichment in enterobactin biosynthesis and related metabolic pathways. In contrast, differences in the gut eukaryotic virome were limited, and no significant enrichment of fecal *Herpesviridae* was detected. Virome perturbations predominantly occurred at the bacteriophage level, featuring shifts in predicted bacterial host assignment from commensal bacteria toward opportunistic pathogen-associated taxa and an increased inferred proportion of temperate phages. Exploratory cross-kingdom analysis suggested associations involving *Escherichia* and three related phage features.

**Conclusion:**

Gut dysbiosis in ARN was associated with *Enterobacteriaceae*-related bacterial remodeling and phage alterations. These findings highlight an ARN-associated bacteriome-phage alteration pattern that warrants validation in larger independent cohorts.

## 1 Introduction

Acute retinal necrosis (ARN) is a severe infectious panuveitis mainly associated with neurotropic α-herpesviruses, including varicella-zoster virus (VZV) as well as herpes simplex virus types 1 and 2 (HSV-1/2) (Wong et al., 2013; Takase et al., 2015). The disease progresses rapidly and is typically characterized by extensive retinal necrosis, occlusive vasculitis, and severe intraocular inflammation. Even with prompt antiviral therapy, patients frequently suffer poor visual outcomes due to secondary retinal detachment (Kalogeropoulos et al., 2024; Wang et al., 2024). α-herpesviruses can establish long-term latency in sensory ganglia after primary infection, with their reactivation being intimately linked to impaired virus-specific immune surveillance. VZV cell-mediated immunity serves as a pivotal defense against symptomatic reactivation, yet this targeted T-cell response progressively diminishes with age (Weinberg et al., 2017). HSV-specific CD8^+^ and CD4^+^ T cells can also be continuously recruited to and reside within sensory ganglia to mediate immune surveillance and prevent viral reactivation (St Leger et al., 2021). IL-1β can trigger HSV-1 reactivation by inducing neuronal hyperexcitability, suggesting that inflammatory signaling itself can lower the threshold for maintenance of viral latency (Cuddy et al., 2020).

The gut microbiome is an integral component in maintaining host immune homeostasis. The gut microbiota can modulate interferon responses and distal antiviral immunity, thereby influencing the outcomes of viral infections (Wirusanti et al., 2022). Meanwhile, gut bacteriophages can influence bacterial community composition and function through lysis, lysogeny, and horizontal gene transfer (Mahmud et al., 2024). Furthermore, phage-derived nucleic acids can exacerbate mucosal inflammation by inducing IFN-γ-related responses via TLR9, suggesting that phages may participate in host immune regulation (Gogokhia et al., 2019). In addition, the gut microecology interacts bidirectionally with neuroendocrine systems such as the hypothalamic pituitary adrenal (HPA) axis, suggesting that its influence may extend to the regulation of systemic inflammation and stress-related signaling (Farzi et al., 2018). Although the gut microbiome plays an important role in host antiviral immunity and inflammatory regulation, its associated alterations in ARN remain unclear.

However, current studies on ARN have mainly focused on intraocular etiological diagnosis and local clinical manifestations, leaving a systematic understanding of the associated systemic host factors largely unexplored. Against this background, the present study integrated 16S rRNA sequencing and viral metagenomics of fecal samples to systematically profile the gut bacterial and viral communities in ARN patients, and further integrated cross-kingdom association analysis to assess whether ARN is accompanied by disease-related gut microbial remodeling.

## 2 Methods

### 2.1 Study population

This study included 10 newly diagnosed patients with acute retinal necrosis (ARN group) who were recruited from the Department of Ophthalmology of the Second Hospital of Jilin University from July 2022 to June 2024, together with 10 healthy individuals matched for age and sex (N group). All ARN cases fulfilled the diagnostic criteria established by the American Uveitis Society (AUS) (Holland, 1994). Patients were included in the ARN group if they met the following criteria: (1) patient in the active stage of disease; (2) no history of antibiotics, corticosteroids, immunosuppressants, or other related medications within 3 months before fecal sample collection; (3) no active infectious disease, or immune-related disease. The inclusion criteria for the N group followed items (2) and (3) as listed above. All ARN patients were recruited in the outpatient setting and had not been hospitalized before fecal sample collection. Fecal samples from ARN patients were collected before the initiation of systemic or local antiviral therapy. No ARN patient received antiglaucoma therapy before baseline IOP assessment. All participants gave written informed consent. The study followed the Declaration of Helsinki and was approved by the Medical Ethics Committee of the Second Hospital of Jilin University (No. 2025-061). Aqueous humor PCR was performed in all 10 ARN cases, and all were positive for VZV. Demographic and baseline clinical characteristics are summarized in Table 1.

**Table 1:** Baseline characteristics of the study population.

Characteristic	ARN (*n* = 10)	*N* (*n* = 10)
Age (year)	54.4 ± 10.70	46.6 ± 15.14
Sex (male/female)	4/6	4/6
NLR	2.37 ± 0.86	/
Initial vision, logMAR	1.31 ± 0.74	/
Initial intraocular pressure (mmHg)	13.50 (12.35, 17.00)	/
Retinal detachment (%)	60%	/
Arteritis involving large retinal artery (%)	60%	/

ARN, acute retinal necrosis; NLR, neutrophil-to-lymphocyte ratio; logMAR, logarithm of the minimum angle of resolution. Data are presented as mean ± SD, median (interquartile range), or percentage (%).

### 2.2 16S rRNA sequencing of gut bacteriome

Fecal DNA was isolated with the OMEGA Soil DNA Kit. We amplified the V3 V4 region of the bacterial 16S rRNA gene using primers 338F (5′-ACTCCTACGGGAGGCAGCA-3′) and 806R (5′-GGACTACHVGGGTWTCTAAT-3′). After PCR product recovery and quantification, libraries were prepared with the TruSeq Nano DNA LT Library Prep Kit and sequenced in a paired-end format on an Illumina platform. The resulting reads were analyzed in QIIME 2 (v 2022.11) (Bolyen et al., 2019). Primer trimming was carried out with cutadapt, followed by denoising, paired-read joining, and chimera filtering with DADA2 (Callahan et al., 2016), which generated amplicon sequence variants (ASVs) and the corresponding abundance table. Taxonomic assignment of ASVs was then completed using the Greengenes2 database (v 2022.10) (McDonald et al., 2024).

### 2.3 Viral metagenomic sequencing and sequence annotation

Viral metagenomic sequencing was performed as previously described (Liu et al., 2025). Fecal samples were snap-frozen in liquid nitrogen, homogenized, and suspended in PBS. After centrifugation, the resulting supernatant was filtered through a 0.45-μm membrane to deplete eukaryotic cells and bacterial-sized particles. Viral DNA was then purified with the DNeasy PowerSoil Pro Kit. Libraries were prepared using the TruSeq Nano DNA LT Kit (Illumina) and sequenced on the Illumina NovaSeq platform to obtain 150-bp paired-end shotgun reads. Raw sequencing data have been deposited in the NCBI database under BioProject accession number [PRJNA1225217].

Adapter sequences and low-quality bases were removed from raw reads using cutadapt and fastp, respectively (Chen et al., 2018). The resulting clean reads were mapped to the NCBI Viral RefSeq genome database to screen for candidate viral sequences. These sequences were then assembled using metaSPAdes (Nurk et al., 2017), followed by dereplication with MMseqs2 in linclust mode (Steinegger and Söding, 2017). The resulting nonredundant contigs were then searched against the NCBI nucleotide database with BLASTN, and taxonomic labels were assigned according to the lowest common ancestor algorithm. Contigs annotated as viral were retained for subsequent viral community composition analysis. Viral sequences were further identified and quality assessed using VIBRANT, VirSorter2, and CheckV (Kieft et al., 2020; Guo et al., 2021; Nayfach et al., 2021). Gene prediction was performed employing TransGeneScan (Nayfach et al., 2021), and viral gene abundances were normalized by calculating transcripts per million (TPM) values via minimap2 (Li, 2018).

### 2.4 Phage host prediction, lifestyle classification, and AMG annotation

Eukaryotic and prokaryotic viral sequences were distinguished using IPEV, a tool that achieves cross-kingdom identification by integrating the relative distances and frequency features of trinucleotide pairs through a two-dimensional convolutional neural network (2D-CNN) (Yin et al., 2024). To limit bias from short viral fragments with low coverage, only prokaryotic viral contigs exceeding 500 bp were kept for downstream analysis. Phage-related features were subsequently annotated with the PhaBOX platform (Shang et al., 2023). Specifically, PhaMer was used for phage identification, PhaTYP for lifestyle prediction, PhaGCN for taxonomic classification, and CHERRY for host prediction.

To identify potential bacteriophage features and annotate auxiliary metabolic genes (AMGs), prokaryotic viral contigs exceeding 1,000 bp in length were analyzed utilizing VIBRANT (Kieft et al., 2020). The software automatically predicted open reading frames (ORFs), and the translated protein sequences were searched against the KEGG, Pfam, and VOG databases. AMGs encoded by phage sequences were summarized according to the default filtering criteria implemented in the software and the KEGG pathway annotation results. Phage lifestyle, bacterial host assignment, and AMG annotations were treated as computationally inferred features and were not experimentally validated in this study.

### 2.5 Statistical analysis

All statistical analyses were carried out in R version 4.4.1. Group comparisons for continuous variables were performed with the Wilcoxon rank-sum test. α and β diversity metrics were used to evaluate within-sample diversity and between-group community variation, respectively. β diversity was displayed by principal coordinate analysis (PCoA) and assessed with permutational multivariate analysis of variance (PERMANOVA).

Differential bacterial taxa were examined using ANCOM-BC with multiple-testing correction. LEfSe was applied as a complementary effect-size-based approach to characterize taxa contributing to group separation, with thresholds of *p* < 0.05 and LDA score > 2.0. Differential viral taxa were also screened using LEfSe with the same thresholds. Variations in predicted bacterial functional pathways and putative phage AMG-associated pathways were analyzed using linear models in MaAsLin2, with adjusted *p* values used for multiple-testing correction. The overall structural concordance between bacterial and phage communities was evaluated via Procrustes analysis. Associations among phages, bacteria, and functional pathways were assessed using Spearman’s rank correlation, and correlation *p*-values were adjusted for multiple testing. A correlation-based cross-kingdom association network was constructed using thresholds of adjusted *p* < 0.05 and |*r*| > 0.6.

Given the pilot nature and limited sample size of this study, analyses beyond the primary group-level comparisons were considered exploratory. Specifically, subgroup analyses, clinical correlation analyses, cross-kingdom association network analysis, and ROC curve analysis were performed to identify potential microbiome-related patterns associated with ARN.

## 3 Results

### 3.1 Differential analysis of the gut bacteriome in ARN patients

**Figure 1:**
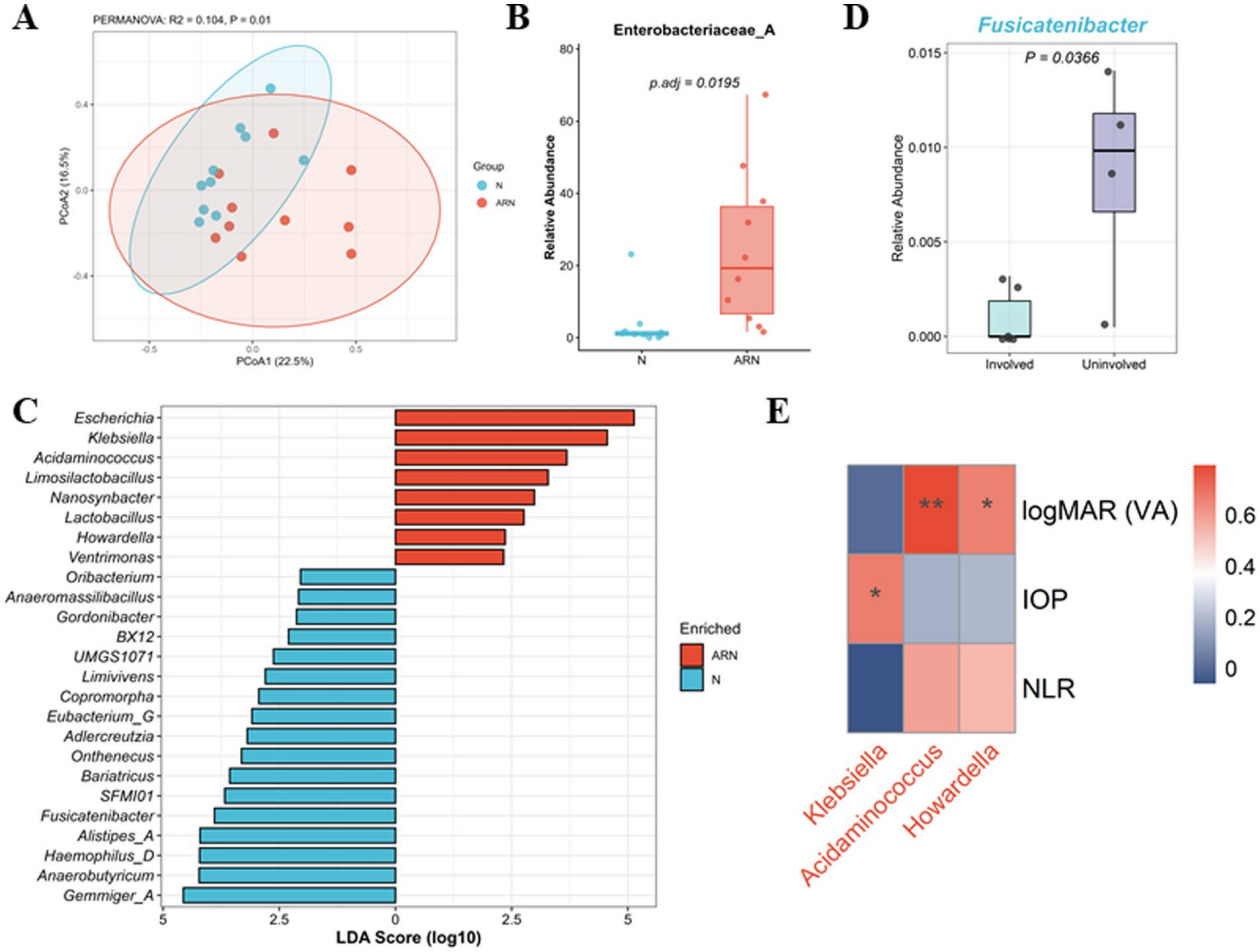
Gut bacteriome remodeling and exploratory clinical associations in ARN patients. **(A)** Principal coordinate analysis of the gut bacterial community. **(B)** Relative abundance of the family *Enterobacteriaceae_A* analyzed by ANCOM-BC with multiple-testing correction. **(C)** Genus-level discriminatory bacterial taxa described by LEfSe analysis. **(D)** Exploratory subgroup analysis showing the relative abundance of *Fusicatenibacter* in ARN patients stratified by the presence or absence of vasculitis involving the major retinal arteries. **(E)** Exploratory correlation heatmap between discriminatory bacterial genera and clinical parameters. *p < 0.05; **p < 0.01.

α diversity metrics, including richness, evenness, and the Shannon index, were comparable between the ARN and N groups (Supplementary Figure S1A). PCoA of Bray-Curtis distances indicated a significant difference in overall community composition between the two groups (PERMANOVA, *p* = 0.01, *R*^2^ = 0.104; Figure 1A). Sample-level 16S sequencing metrics, abundance data, and diversity outputs are provided in Supplementary Table S1.

At the family level, ANCOM-BC identified *Enterobacteriaceae_A* as significantly increased in the ARN group after multiple-testing correction (*p.adj* = 0.0195; Figure 1B). At the genus level, no taxonomically resolved genus remained significant after correction in the ANCOM-BC analysis. LEfSe was therefore used as a complementary effect-size-based approach to describe genus-level discriminatory patterns, while correction-validated genus-level differences were not inferred from this analysis (Figure 1C). This analysis described 25 discriminatory genera. Compared to the N group, the ARN group exhibited an enrichment of 8 genera, including opportunistic pathogens such as *Escherichia* and *Klebsiella*, and a depletion of 17 genera, including commensal bacteria such as *Fusicatenibacter* and *Anaerobutyricum*. Bacterial taxonomic differential abundance outputs from ANCOM-BC and LEfSe are provided in Supplementary Table S3.

In exploratory subgroup analysis, *Fusicatenibacter* showed a lower relative abundance in patients with vasculitis involving the major retinal arteries (Figure 1D), whereas no differential genera were observed in the subgroup analysis according to the presence or absence of retinal detachment. Exploratory clinical correlation analysis further suggested potential associations between specific differential genera and clinical indices. *Klebsiella* was positively correlated with intraocular pressure (IOP), while *Acidaminococcus* and *Howardella* exhibited positive correlations with logMAR (Figure 1E).

### 3.2 Differential analysis of gut bacterial metabolic functions in ARN patients

**Figure 2:**
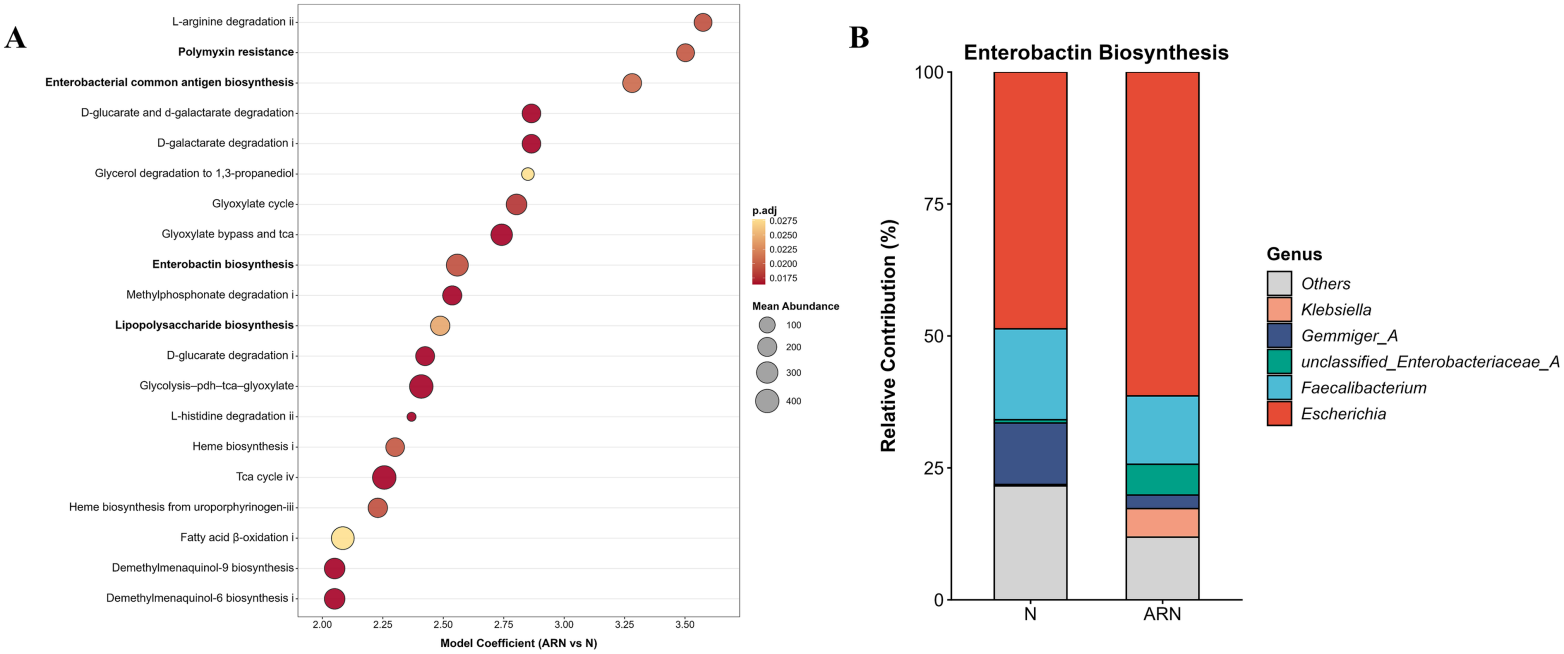
Predicted bacterial pathway profiles in ARN patients. **(A)** Top 20 FDR-ranked differential predicted bacterial pathways between the ARN and N groups evaluated by MaAsLin2. Pathways were selected according to FDR and ordered by model coefficients. Dot size indicates mean abundance, and color indicates FDR. **(B)** Taxonomic contribution of bacterial genera to the enterobactin biosynthesis pathway.

Based on bacterial functional predictions by PICRUSt2, MaAsLin2 analysis identified 42 predicted pathways showing differential abundance (FDR < 0.05 and |Model_coef| ≥ 2), all of which were enriched in the ARN group. Figure 2A displays the top 20 FDR-ranked pathways, ordered by model coefficient to highlight effect-size differences. These pathways included core carbon metabolism, enterobactin biosynthesis, enterobacterial common antigen biosynthesis, the superpathway of lipopolysaccharide biosynthesis, and polymyxin resistance. Further taxonomic contribution analysis suggested that the predicted abundance of this pathway was largely attributable to *Enterobacteriaceae*-related taxa, notably *Escherichia*, unclassified *Enterobacteriaceae_A*, and *Klebsiella* (Figure 2B). Complete bacterial predicted functional pathway outputs are provided in Supplementary Table S5.

### 3.3 Characterization of the gut virome in ARN patients

**Figure 3:**
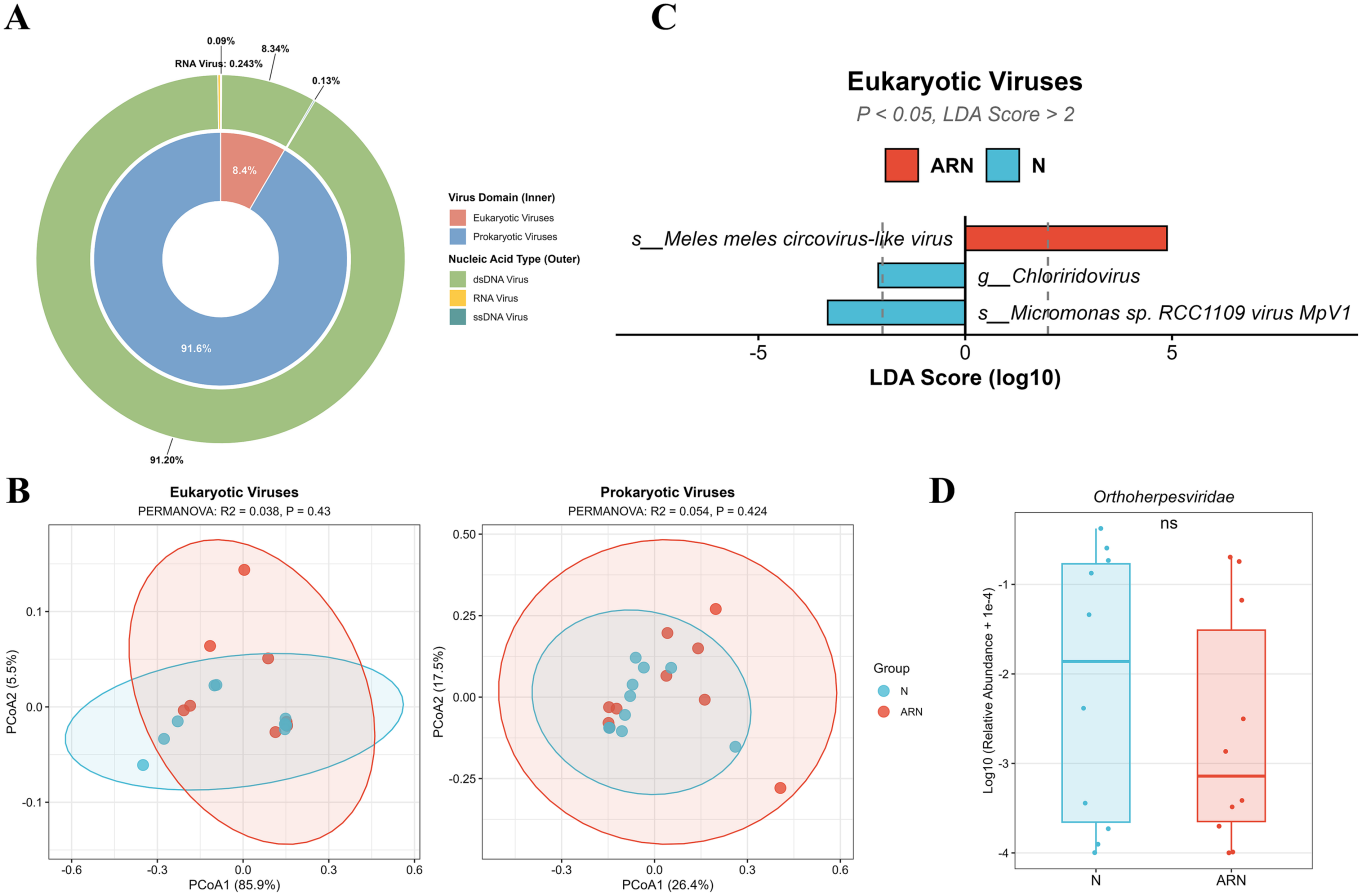
Fecal DNA virome profile in ARN patients. **(A)** Composition of fecal viral sequences according to viral domain and nucleic acid type. **(B)** Principal coordinate analysis of the overall community structures for eukaryotic and prokaryotic viruses. **(C)** Differentially abundant eukaryotic viral taxa identified by LEfSe analysis. **(D)** Relative abundance of fecal Orthoherpesviridae-associated sequences between the N and ARN groups. Orthoherpesviridae corresponds to the family formerly referred to as Herpesviridae in earlier taxonomy.

Based on viral metagenomic sequencing, a total of 23,139 viral contigs were assembled. Viral domain classification using IPEV, in combination with gene prediction, revealed that prokaryotic and eukaryotic viral sequences accounted for 91.6 and 8.4% of the total, respectively, with both viral realms predominantly composed of dsDNA viruses (Figure 3A). Diversity analysis showed no significant differences in the overall structure of either the eukaryotic virome or the prokaryotic virome between the ARN and N groups (Supplementary Figure S1B; Figure 3B). Viral metagenomic sequencing summaries and virome diversity outputs are provided in Supplementary Table S2.

Differential analysis of eukaryotic viral taxa revealed that the circovirus-related species *Meles meles circovirus-like virus* was significantly enriched in the ARN group, whereas the species *Micromonas* sp. RCC1109 virus MpV1 and the genus *Chloriridovirus* were significantly depleted (Figure 3C). Low-abundance Herpesvirus-associated sequences were detected in a subset of fecal samples, with a mean relative abundance below 0.2%. Within the fecal virome dataset, *Herpesviridae*-associated sequences did not show significant enrichment in the ARN group (Figure 3D). Supporting outputs for eukaryotic viral differential analysis are provided in Supplementary Table S4.

### 3.4 Differential analysis of the gut virome in ARN patients

**Figure 4:**
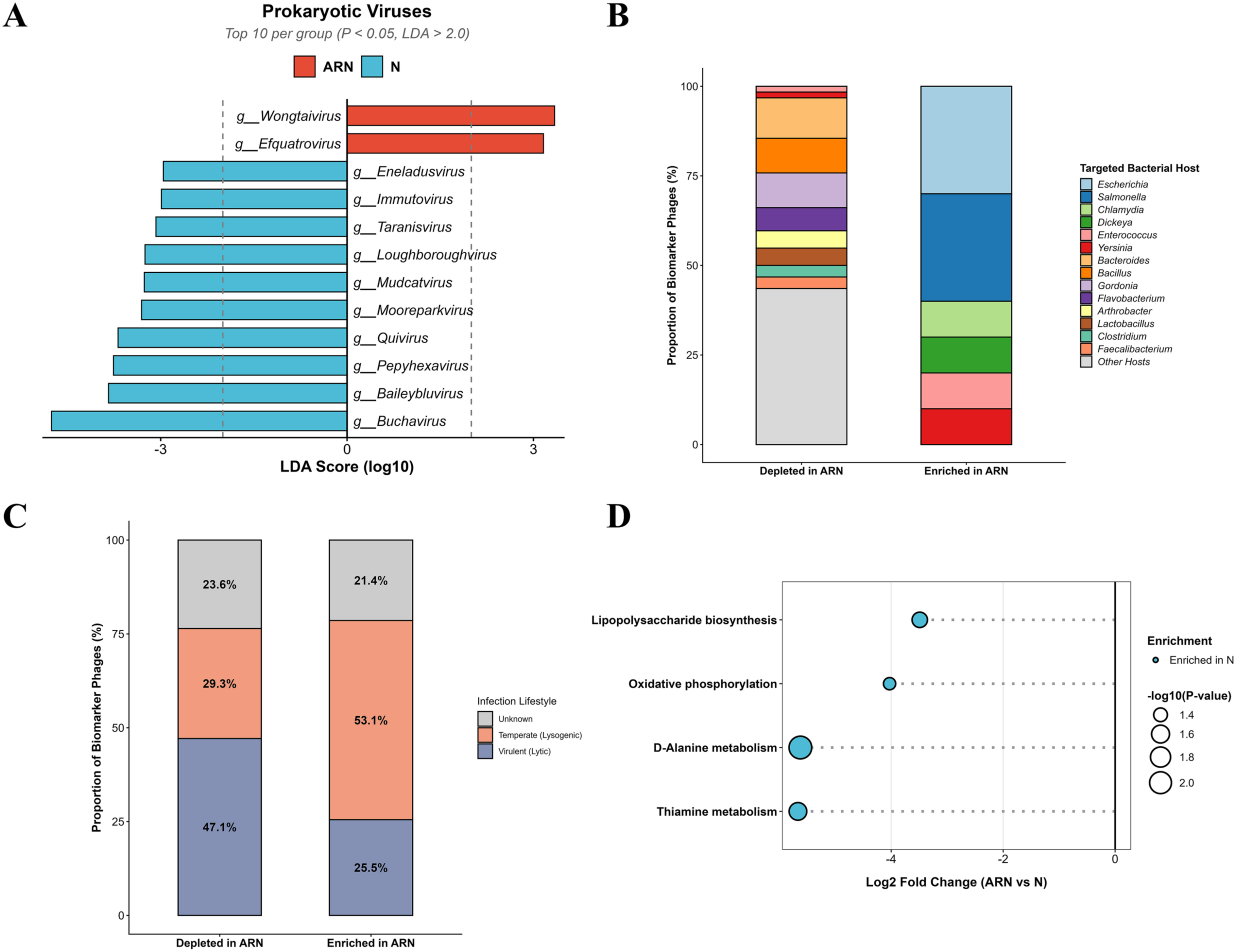
Characteristics of differential gut phages in ARN patients. **(A)** Differentially abundant phage genera identified by LEfSe analysis. **(B)** Bacterial host annotations of the differential phage species depleted or enriched in the ARN group. **(C)** Lifestyle (temperate or virulent) distribution of the differential phage species. **(D)** Phage auxiliary metabolic gene (AMG)-associated pathways evaluated by MaAsLin2.

Compared to eukaryotic viruses, inter-group differences were predominantly concentrated at the phage level. Using LEfSe analysis (*p* < 0.05, LDA > 2), we identified ARN-associated differential phages (Figure 4A). At the family level, the relative abundances of *Straboviridae* (LDA = 4.08), *Salasmaviridae* (LDA = 3.69), and *Suolaviridae* (LDA = 2.04) were significantly reduced in the ARN group. At the genus and species levels, 37 differential phage genera (2 enriched and 35 depleted in the ARN cohort) and 73 differential species (10 enriched and 63 depleted) were identified, respectively.

Host assignment of the differential phages at the species level revealed that the phages depleted in the ARN group were primarily assigned to gut commensals, such as *Bacteroides* and *Faecalibacterium*. Conversely, the ARN-enriched phages were more frequently assigned to opportunistic pathogen associated taxa, including *Escherichia*, *Salmonella*, and *Enterococcus* (Figure 4B). Inferred lifestyle analysis further indicated that the differential phages depleted in the ARN group were mainly virulent (accounting for 47.1%), whereas the proportion of temperate phages increased to 53.1% among the differential phages enriched in the ARN group (Figure 4C). Supporting outputs are provided in Supplementary Table S4.

In addition, exploratory analysis of phage AMG-associated pathways showed nominal reductions in D-alanine metabolism, lipopolysaccharide biosynthesis, thiamine metabolism, and oxidative phosphorylation in the ARN group (Figure 4D; Supplementary Table S6). These AMG-associated signals did not remain significant after FDR correction.

### 3.5 Cross-kingdom associations between gut phages and bacteria in ARN patients

To evaluate the overall concordance between the bacteriome and the phageome, we first performed a Procrustes analysis. The results revealed no significant correspondence between the community structures of phages and bacteria (Supplementary Figure S1C). After filtering out taxa with an average relative abundance below 0.01%, Spearman correlation analysis was conducted on the differential phages (at the species level) and differential bacteria (at the genus level) identified by LEfSe. A total of three phage-bacterium pairs showed positive correlations together with predicted host correspondence (Table 2), namely *Enterobacteria phage mEp237* and *Escherichia*, *Wanchaivirus HK106* and *Escherichia*, as well as *Wongtaivirus ECP1* and *Escherichia*. These pairs showed parallel enrichment in the ARN group.

**Table 2:** Representative phage-bacterium associations in the gut microbiome.

Host (bacteria)	Virus (species)	Lifestyle	|*r*|	*p.adj*
*Escherichia*↑	*Enterobacteria phage mEp237*↑	Temperate	0.67	0.001
	*Wanchaivirus HK106*↑	Temperate	0.66	0.002
	*Wongtaivirus ECP1*↑	Temperate	0.52	0.017

**Figure 5:**
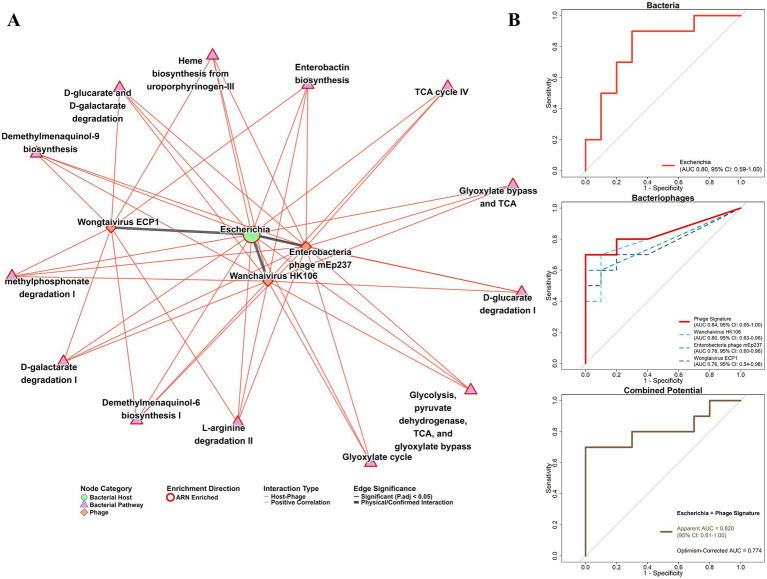
Exploratory *Escherichia*-phage association pattern and descriptive ROC curves in ARN patients. **(A)** Localized cross-kingdom association network involving *Escherichia*, related phage features, and predicted bacterial functional pathways. **(B)** Descriptive ROC curves for *Escherichia*, the three-phage feature set, and the combined exploratory model.

We further constructed a cross-kingdom correlation network (*p.adj* < 0.05, |*r*| > 0.6) to visualize associations among gut phages, bacteria, and predicted metabolic functions (Figure 5A). The network highlighted a localized association pattern involving *Escherichia*, related phage features, and predicted bacterial functional pathways. Specifically, *Escherichia* and three correlated phage features (*Enterobacteria phage mEp237*, *Wanchaivirus HK106*, and *Wongtaivirus ECP1*) exhibited positive correlations with several predicted bacterial metabolic pathways, including enterobactin biosynthesis, and D-glucarate degradation I.

We further used ROC curves as a descriptive summary of the apparent group separation associated with *Escherichia* and the three correlated phage features (Figure 5B). *Escherichia* showed an apparent AUC of 0.800 (95% CI: 0.593–1.000), whereas the three-phage feature set showed an apparent AUC of 0.840 (95% CI: 0.652–1.000). A combined model integrating *Escherichia* and the three phage features yielded an apparent AUC of 0.820 (95% CI: 0.612–1.000). After bootstrap-based internal validation, the optimism-corrected AUC was 0.774. Supporting outputs for the cross-kingdom network and exploratory ROC analysis are provided in Supplementary Table S7.

## 4 Discussion

In this cross-sectional pilot study, we systematically characterized alterations in the gut bacteriome and virome of patients with active ARN. Gut dysbiosis associated with ARN did not manifest as a pronounced expansion of eukaryotic viruses, particularly herpesviruses, in the gut. Instead, the disease state was accompanied mainly by bacteriome remodeling and phage-level perturbations. Further integrative analysis indicated that these microecological shifts tended to represent a localized cross-kingdom association pattern centered on *Enterobacteriaceae*, rather than a global, synchronous imbalance of the gut microbiome.

Alterations in the gut bacterial community of ARN patients were primarily characterized by an increase in *Enterobacteriaceae*-related taxa, accompanied by a reduction in certain commensal anaerobes. Similar dysbiotic features, including depletion of commensal or short-chain fatty acid-producing taxa and enrichment of inflammation-associated bacteria, have been described across several non-infectious or immune-mediated uveitis phenotypes (Agrawal et al., 2025). Therefore, the *Enterobacteriaceae*-related expansion and loss of certain commensal anaerobes observed in ARN may partly reflect a broader inflammation-associated gut microbial pattern rather than an ARN-specific bacterial signature. In this context, the exploratory associations of *Klebsiella*, *Acidaminococcus*, and *Howardella* with ocular clinical indices may reflect disease-state inflammatory dysbiosis rather than direct microbial effects on IOP or visual outcome (Baldelli et al., 2021; Agrawal et al., 2025). Previous studies have indicated that the expansion of *Enterobacteriaceae* is frequently regarded as a hallmark of inflammation-associated gut dysbiosis, with specific members possessing pathobiont properties capable of exacerbating host inflammatory responses (Baldelli et al., 2021). In contrast, *Fusicatenibacter saccharivorans* is depleted in patients with active ulcerative colitis and has been reported to induce the anti-inflammatory cytokine IL-10 (Takeshita et al., 2016). Furthermore, *Anaerobutyricum hallii* (formerly *Eubacterium hallii*) can produce butyrate from lactate and acetate and is considered an important commensal anaerobe for maintaining gut metabolic homeostasis (Shetty et al., 2018). Moreover, previous studies indicate that gut commensals are fundamentally involved in maintaining the priming of host basal antiviral immunity. Erttmann et al. found that gut commensals can sustain peripheral cGAS-STING-dependent basal type I interferon signaling by releasing DNA-containing membrane vesicles, thereby enhancing host resistance to systemic viral infections (Erttmann et al., 2022). Oh et al. further demonstrated that antibiotic-induced dysbiosis leads to elevated local IL-33 levels and delayed HSV-2 clearance, suggesting that dysbiosis itself may impair anti-herpesvirus immunity (Oh et al., 2016). Together, these findings suggest that active ARN is accompanied by an altered gut microbial pattern characterized by the expansion of Enterobacteriaceae-related taxa and the depletion of selected commensal anaerobes. Although previous studies have linked gut commensals to basal antiviral immune priming, the present cross-sectional data do not establish whether these microbial differences preceded ARN or influenced antiviral immune responses. Longitudinal studies integrating immune profiling and functional validation will be required to clarify the biological significance of these microbial alterations.

Although all ARN cases were confirmed as VZV-positive by aqueous humor PCR, *Herpesviridae*-associated sequences remained low in fecal viromes and were not enriched in the ARN group. These parallel findings distinguish the ocular viral diagnosis from the fecal virome profile and indicate that gut virome alterations in active ARN were concentrated mainly in the bacteriophage. Previous studies have shown that the human gut virome is predominantly composed of phages, with high interindividual specificity and a large proportion of unannotated sequences (Shkoporov et al., 2019). In disease states, such as inflammatory bowel disease, gut virome dysbiosis frequently manifests as rearrangements in both phage composition and lifestyles (Clooney et al., 2019). In the current study, ARN-associated bacteriophage alterations were primarily characterized by a concurrent shift in host-targeting preferences and lifestyles. Specifically, we observed a reduction in phages associated with commensal bacteria, an increase in phages associated with opportunistic pathogens, and a higher proportion of temperate phages among the enriched phages. From an ecological perspective, the Piggyback-the-Winner model proposes that temperate strategies are more likely to predominate when host density increases and environmental stress intensifies (Knowles et al., 2016). Additionally, host-derived quorum sensing signals can be sensed by phages, directly influencing their lysis-lysogeny decisions (Silpe and Bassler, 2019). Taken together, these observations suggest that phage-level changes in ARN may occur alongside altered bacterial community structure.

The predicted bacterial pathway profiles and putative phage AMG-associated profiles did not show parallel patterns in ARN patients. In healthy controls, putative phage AMG-associated pathways were more abundant, a pattern consistent with previous evidence that viral AMGs can provide insight into viral community functional potential (Kieft et al., 2020). Meanwhile, viral lifestyle serves as an important factor influencing the composition of AMG. Virulent phages generally harbor a higher diversity of AMGs, whereas temperate phages are more likely to retain functions associated with long-term host persistence (Luo et al., 2022). Conversely, predicted bacterial pathway profiles in the ARN group showed increased abundance of bacterial metabolic pathways. Enterobactin is a high-affinity siderophore produced by Enterobacteriaceae. Beyond its role in facilitating iron acquisition, enterobactin can inhibit host myeloperoxidase activity, thereby enhancing the survival advantage of pathogenic *Escherichia coli* in the inflammatory gut (Singh et al., 2015). Combined with the expansion of *Escherichia* and *Klebsiella* in the ARN group, the enrichment of the enterobactin biosynthesis pathway may reflect *Enterobacteriaceae*-related adaptive metabolic potential under inflammatory conditions. Cross-kingdom association analysis further suggested that these bacterial and phage features were correlated with predicted bacterial functional pathways within a localized association pattern centered on *Escherichia* and related phage features. Thus, ARN-associated gut dysbiosis may involve Enterobacteriaceae-related bacterial remodeling together with accompanying phage-level alterations.

This study has several limitations. First, the cohort size was small, including only 10 ARN patients and 10 healthy controls, which limited statistical power and increased the risk of unstable estimates, false-positive associations, and overfitted discriminative signals in high-dimensional bacteriome and virome analyses. Therefore, subgroup analyses, clinical correlations, cross-kingdom networks, phage-host associations, and ROC-based modeling should be considered exploratory and require validation in larger independent cohorts. External validation and formal sensitivity analyses using alternative analytical thresholds were not performed. Second, although recent exposure to antibiotics, corticosteroids, and immunosuppressants was excluded, all ARN patients were outpatients, and fecal samples were collected before systemic or local antiviral therapy, residual confounding from diet, fasting status, bowel symptoms, BMI, metabolic status, smoking, alcohol exposure, lifestyle factors, and psychological stress could not be fully controlled. Third, the cross-sectional design precludes temporal or causal inference. Finally, stool viral metagenomic profiling, PICRUSt2-based bacterial pathway prediction, phage lifestyle inference, host assignment, AMG annotation, and correlation-based network analysis were computationally inferred and require further biological validation. 16S rRNA sequencing also has limited species-level resolution and does not directly assess bacterial activity or metabolite production.

In summary, this exploratory cross-sectional study suggests that active ARN is associated with a gut microecological pattern characterized by *Enterobacteriaceae*-related bacterial remodeling and a localized *Escherichia*–phage association pattern, rather than overt enrichment of fecal *Herpesviridae*. These findings highlight bacteriome–phage alterations associated with the ARN disease state and provide a basis for future longitudinal studies examining whether these microbial associations are temporally or functionally related to infectious uveitis. Given the small cohort size and the high-dimensional analytical framework, these observations should be regarded as preliminary and require confirmation in larger independent cohorts.

## Data Availability

The viral metagenomic sequencing raw data reported in this paper have been deposited in the NCBI BioProject database under accession number PRJNA1225217. The 16S rRNA sequencing raw data reported in this paper have been deposited in the NCBI BioProject database under accession number PRJNA1446193.
