# Supplementary material for: Remodeling of gut bacteriome and virome in acute retinal necrosis: expansion of Enterobacteriaceae-related taxa
Source: Front Microbiol. 2026 Jun 29;17:1848524. doi: 10.3389/fmicb.2026.1848524 (PMC13357178; doi:10.3389/fmicb.2026.1848524)
Supplement: Supplementary file 1 [file Supplementary_file_1.DOCX]

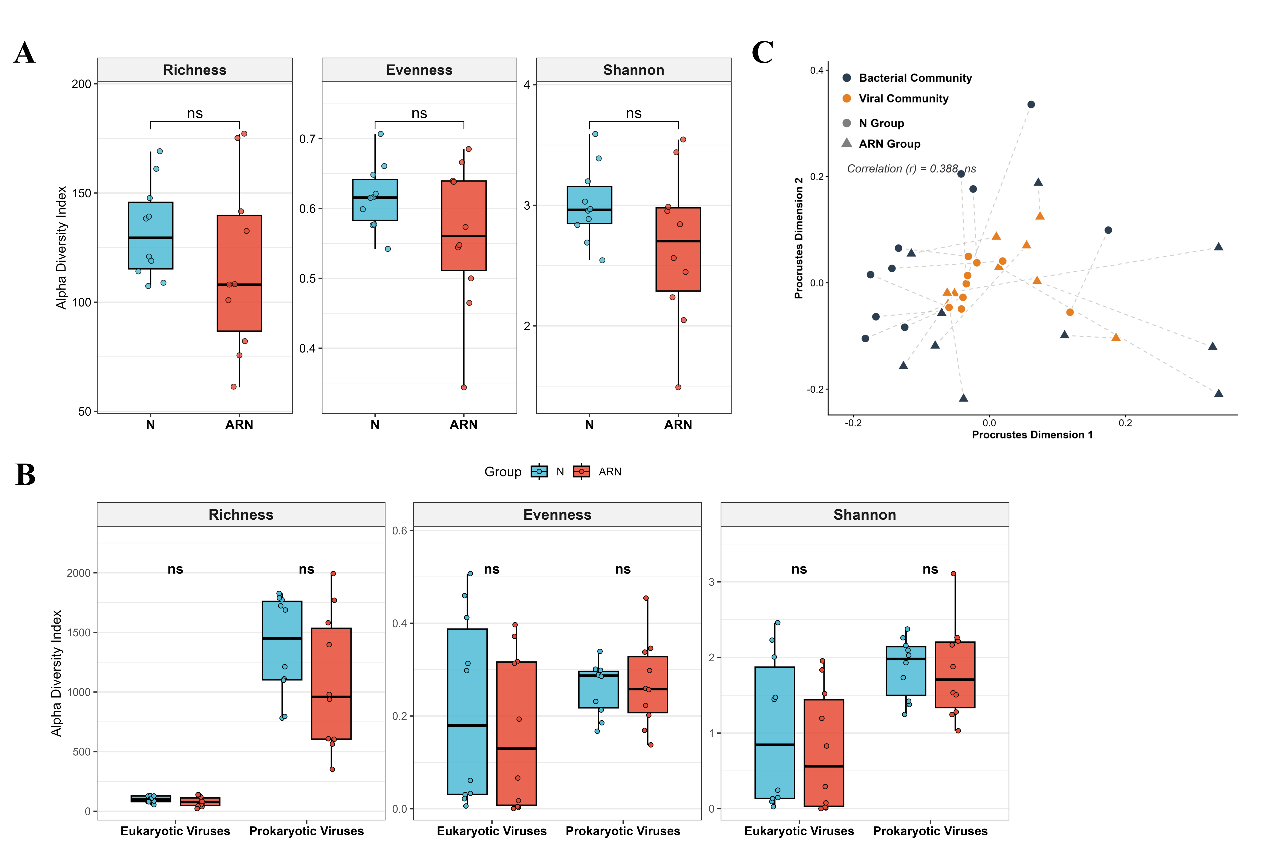


**Supplementary Figure 1.** Alpha diversity and global bacteriome-phageome concordance analyses.

(A) α diversity indices (Richness, Evenness, and Shannon) of the gut bacterial community. (B) α-diversity indices of fecal eukaryotic and prokaryotic viral communities. (C) Procrustes analysis evaluating the overall structural concordance between the gut bacterial community and fecal viral community.
